# Interaction between Macrophages and Nanoparticles: In Vitro 3D Cultures for the Realistic Assessment of Inflammatory Activation and Modulation of Innate Memory

**DOI:** 10.3390/nano11010207

**Published:** 2021-01-15

**Authors:** Benjamin J. Swartzwelter, Alessandro Verde, Laura Rehak, Mariusz Madej, Victor. F. Puntes, Anna Chiara De Luca, Diana Boraschi, Paola Italiani

**Affiliations:** 1 Institute of Biochemistry and Cell Biology, National Research Council, 80131 Napoli, Italy; swartzwe@colorado.edu (B.J.S.); alessandro.verde@ibbc.cnr.it (A.V.); mariusz.madej@ocello.nl (M.M.); annachiara.deluca@ibbc.cnr.it (A.C.D.L.); 2 Athena Biomedical Innovations, 00100 Roma, Italy; laurarehak@gmail.com; 3 Institut Català de Nanociència i Nanotecnologia (ICN2), CSIC and The Barcelona Institute of Science and Technology (BIST), Campus UAB, 08193 Bellaterra, Barcelona, Spain; victor.puntes@icn2.cat; 4 Stazione Zoologica Anton Dohrn, 80121 Napoli, Italy

**Keywords:** monocytes, macrophages, gold nanoparticles, in vitro models, innate immunity, inflammation, innate memory, 2D cultures, 3D cultures

## Abstract

Understanding the modes of interaction between human monocytes/macrophages and engineered nanoparticles is the basis for assessing particle safety, in terms of activation of innate/inflammatory reactions, and their possible exploitation for medical applications. In vitro assessment of nanoparticle-macrophage interaction allows for examining the response of primary human cells, but the conventional 2D cultures do not reproduce the three-dimensional spacing of a tissue and the interaction of macrophages with the extracellular tissue matrix, conditions that shape macrophage recognition capacity and reactivity. Here, we have compared traditional 2D cultures with cultures on a 3D collagen matrix for evaluating the capacity gold nanoparticles to induce monocyte activation and subsequent innate memory in human blood monocytes in comparison to bacterial LPS. Results show that monocytes react to stimuli almost in the same way in 2D and 3D cultures in terms of production of TNFα and IL-6, but that notable differences are found when IL-8 and IL-1Ra are examined, in particular in the recall/memory response of primed cells to a second stimulation, with the 3D cultures showing cell activation and memory effects of nanoparticles better. In addition, the response variations in monocytes/macrophages from different donors point towards a personalized assessment of the nanoparticle effects on macrophage activation.

## 1. Introduction

The interaction of engineered nanoparticles (NPs) with the immune system is a key element both in the assessment of nanotoxicity and in the development of nanomedicines and nanomedical devices [1,2]. Possible changes in immune functions upon interaction with NPs may affect the defensive capability of exposed organisms, thereby increasing their susceptibility to infections and diseases. The immune mechanisms that are principally involved in the interaction with NPs are those of innate immunity, the ancient highly conserved defensive system shared by all organisms, from plants to humans [3]. In humans, innate immunity is mainly based on the surveillance action of a number of cell types, in particular the mononuclear phagocytes (monocytes and macrophages) that populate every tissue in the body with the task of recognizing and eliminating possible threats [4]. Macrophages are resident in tissues and are deputed to phagocytosis and efferocytosis, being a little reactive to stimulation (e.g., with bacteria) to avoid mounting a potentially destructive inflammatory reaction [5,6]. On the other hand, when a potential danger is identified, macrophages and other tissue cells produce chemotactic factors that attract effector cells from the blood. Monocytes are among these cells, and participate in the effector phase of inflammation/innate defence by efficiently reacting to foreign agents to produce a number of inflammation-related factors, including both inflammatory cytokines such as TNFα and anti-inflammatory agents able to turn off the reaction when the danger is eliminated [6,7]. Many studies have addressed the possible inflammatory/toxic effects of engineered NPs by examining their interaction with monocytes and macrophages. These studies yielded variable results, and their relevance in predicting effects in human beings is unclear. Many studies have been performed with NPs that were not tested for the presence of endotoxin, a ubiquitous bacterial contaminant that cannot be eliminated by sterilization and that is among the most effective activators of monocytes/macrophages [8], a fact that may lead to misinterpretation of the NP inflammatory effect results. In vivo studies are generally performed in the mouse, an animal that, like human beings, possesses both innate and adaptive immunity but that presents a number of important immune-related differences from humans [9,10]. In vitro studies are largely based on transformed macrophage-like cell lines, which ensure repeatability of results but do not reproduce the behaviour of normal non-tumour cells. Studies with human primary monocytes and monocyte-derived macrophages can mimic several of the conditions of real life, although in many cases they miss the important interaction of these cells with their tissue microenvironment. We have previously established a complex kinetic in vitro model that reproduces the entire course of an inflammatory reaction, as it is experienced by human monocytes that enter an inflamed tissue [11]. With this model, we have described the lack of toxicity/direct inflammatory effect of a number of engineered NPs (Au, Ag, Fe_x_O_y_), used at realistic concentrations, tested for the lack of endotoxin contamination and coated with a biocorona of human serum, as it would happen to NPs entering a human tissue [12,13]. The fact that NPs do not have a direct effect on monocytes/macrophages in inducing inflammation does not exclude that they could have other much subtler effects in modulating their functions. It is known that innate immune cells can develop memory, i.e., reprogram their responses to challenges based on their previous exposure experience [14,15,16,17]. Innate memory aims at making monocytes/macrophages better able to cope with subsequent challenges after an initial exposure and is the most important immune defensive mechanism in plants and invertebrates [18,19,20]. In some cases, however, innate memory can lead to a pathological exacerbation of secondary reactions [21,22,23]. While our understanding of innate memory is still largely incomplete, it is tempting to speculate that engineered NPs might be used for a targeted reprogramming of innate immunity for preventive and therapeutic scopes, such as in vaccination and immunotherapy [24,25,26,27]. Few recent studies show that indeed NPs may prime human monocytes and vertebrate/invertebrate macrophages and induce a modification of their response to a subsequent inflammatory challenge [28,29,30,31,32]. In this study, we have examined the memory-inducing capacity of AuNPs on human primary monocytes to define the most suitable conditions for a realistic evaluation. The choice of AuNPs is based on the fact that they are considered inert and harmless and are therefore largely used in many different clinical applications, raising our interest in examining the possibility that they may nevertheless display immunomodulatory effects. Thus, we have assessed the individual response of cells from six individual donors, to assess the impact of the “immunobiography”, i.e., the previous history of exposure, on monocyte reactivity and memory [33]. We have measured the production of four different types of inflammation-related factors to better understand their mutual balance in the overall response; these are two inflammatory factors (TNFα and IL-6), an alarmin (the chemokine IL-8) and an anti-inflammatory cytokine (IL-1Ra). Lastly, we have compared the cell functions (primary activation, induction of memory, memory response) of monocytes/macrophages cultured in conventional 2D plates with those of cells seeded in a 3D collagen matrix that represents the architecture of a tissue (skin in this case) and its components. The results show that the production of inflammatory factors is not substantially different in cells cultured in 2D vs. 3D, whereas there is a more abundant production of the alarmin and the anti-inflammatory factor in 3D cultures. The results also show the capacity of AuNPs to induce innate memory was evident in 3D cultures, with the effects being strongly donor-dependent.

## 2. Materials and Methods 

### 2.1. Synthesis and Characterization of AuNP

#### 2.1.1. AuNP Synthesis and Purification

Synthesis of AuNPs was conducted using wet chemistry methods as previously described [34]. Briefly, 1 mL of 25 mM HAuCl_4_ was rapidly added to 150 mL of a boiling aqueous solution of sodium citrate 2.2 mM. The formation of AuNPs (~10 nm) occurred in a few minutes, and was followed by growth-inducing steps, consisting of the addition of HAuCl_4_ until reaching the desired NP size. Au NPs of 12 nm diameter were used in this study. Sodium citrate tribasic dihydrate (≥99% purity), gold (III) chloride trihydrate HAuCl_4_·3H_2_O (99.9% purity) were from Sigma-Aldrich, Inc. (St. Louis, MO, USA). All reagents were used as received without further purification, and all glass material was sterilized and dehydrogenated in an oven prior to use. Pyrogen-free milli-Q water was used in the preparation of all solutions. AuNPs were purified by centrifugation to discard byproducts and contaminants, then NPs were resuspended in a solution of 2.2 mM sodium citrate (final pH = 6.4) and stored at 4 °C in the dark.

#### 2.1.2. Nanoparticle Characterization

NP characterisation was performed as previously described [34]. Briefly, STEM (scanning transmission electron microscopy) images were acquired with an FEI Magellan XHR scanning electron microscope (SEM) (FEI, Hillsboro, OR, USA), operated in transmission mode at 20 kV. UV-Vis spectra of AuNPs in sodium citrate were acquired at room temperature using a Shimadzu UV-2400 spectrophotometer (SSI; Kyoto, Japan), reading a spectral range from 300 to 750 nm. The NP hydrodynamic diameter and Z-potential were acquired by dynamic light scattering and laser doppler velocimetry, using a Malvern Zetasizer Nano ZS instrument (Malvern Panalytical Ltd., Malvern, UK) equipped with a light source wavelength of 632.8 nm and a fixed scattering angle of 173°. 

### 2.2. LAL Assay

The endotoxin contamination of the NPs was assessed using the chromogenic Pyrochrome LAL assay (Associates of Cape Cod, Inc.; East Falmouth, MA, USA), according to an optimized protocol that included a number of interference controls [35]. The endotoxin contamination in the AuNPs used in this study was 9.0 EU/mg, which excluded the possibility that endotoxin could activate monocytes in the culture at the NP concentration used (1 µg/mL, corresponding to an endotoxin contamination of 0.009 EU/mL, and 10 µg/mL, corresponding to an endotoxin contamination of 0.09 EU/mL, with monocyte activation detectable above 0.1 EU/mL) [36]. 

### 2.3. Human Monocyte Isolation and Culture

Blood was obtained from healthy donors, upon informed consent and in agreement with the Declaration of Helsinki. The protocol was approved by the Regional Ethics Committee for Clinical Experimentation of the Tuscany Region (Ethics Committee Register n. 14,914 of 16 May 2019). Donors were between 25 and 35 years of age, healthy, non-obese, no smokers, not taking medications. Donors 5 and 6 were female, donors 1 and 3 were of Indo-Aryan ethnicity, and donors 2 and 4–6 were of Caucasian ethnicity. Monocytes were isolated by CD14 positive selection with magnetic microbeads (Miltenyi Biotec, Bergisch Gladbach, Germany) from peripheral blood mononuclear cells (PBMC), obtained by Ficoll-Paque gradient density separation (GE Healthcare, Bio-Sciences AB, Uppsala, Sweden). Monocyte preparations used in the experiments were >95% viable and >95% pure (assessed by trypan blue exclusion and cytosmears). 

Monocytes were cultured in culture medium (RPMI 1640 + Glutamax-I; GIBCO by Life Technologies, Paisley, UK) supplemented with 50 µg/mL gentamicin sulfate (GIBCO) and 5% heat-inactivated human AB serum (Sigma-Aldrich, Inc.). Cells (5 × 10^5^) were seeded in a final volume of 0.5 mL in control wells of 24-well flat-bottom plates (well internal diameter 15.6 mm; Corning^®^ Costar^®^; Corning Inc. Life Sciences, Oneonta, NY, USA), or in wells that contained a round clipping of a 3D collagen matrix (diameter 15.4 mm; stabilized collagen type I from decellularized bovine skin, pore size around 100 μm, thickness 2 mm; clinically used for dermal regeneration; Nevelia^®^, Symathese Biomateriaux, Chaponost, France), obtained with a puncheon tool. Characterisation of monocytes in 3D cultures showed that they can undergo differentiation into macrophages (both spontaneous and CSF-1 mediated) as expected, and express all the phenotypic and functional markers of resting, M1 and M2 macrophages, depending on the applied stimuli (data not shown).

### 2.4. Cell Activation and Induction of Innate Memory

After overnight resting, monocytes were exposed for 24 h to AuNPs (1 µg/mL) or LPS (1 ng/mL; from *E. coli* O55:B5; Sigma-Aldrich, Inc.) or left untreated (control). AuNPs were pre-incubated in 50% human AB serum for 60 min at 37 °C, before addition to monocyte cultures. This procedure allowed for the formation of a physiologically relevant biocorona that prevented particle aggregation in the culture medium [37]. Ion release and reduction of NP size were not detectable after 24 h in culture medium without monocytes, thereby excluding particle dissolution (data not shown) [38]. After supernatant collection, cells were washed and cultured with fresh culture medium for an additional 6 days (one medium change) to allow the extinction of the activation induced by the previous stimulation. After the resting phase, the supernatant was collected, and cells were challenged for an additional 24 h with fresh medium alone or containing a ten-fold higher concentration of LPS (10 ng/mL). All supernatants (after the first stimulation, after the resting phase and after the challenge phase) were frozen at −20 °C for subsequent cytokine analysis. By visual inspection, cell viability and cell number did not substantially change in response to the different treatments.

### 2.5. Cytokine Analysis

The levels of the inflammatory cytokines TNFα and IL-6, of the chemokine IL-8 and of the anti-inflammatory factor IL-1Ra were assessed by ELISA (R&D Systems, Minneapolis, MN, USA). The absorbance of the assay wavelength was measured at 450 nm (subtracting background present at 550 nm) using a Cytation 3 imaging reader (BioTek, Winooski, VT, USA).

### 2.6. Statistical Analysis

Data from cytokine measurements have been analysed using the GraphPad Prism9 software (GraphPad Inc., La Jolla, CA, USA), and are presented in terms of ng/10^6^ plated monocytes. Results are reported as mean ± SD of values from 2–8 replicates from the same donor or as median ± quartiles of values from different donors. The statistical significance of differences is indicated by *p* values, calculated using a paired one-tailed non-parametric Wilcoxon signed-rank test.

## 3. Results

### 3.1. Gold Nanoparticle Characterisation

**Figure 1:**
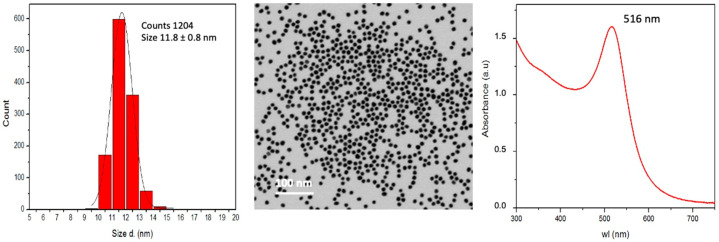
Main characteristics of AuNPs. (**Left**): size distribution by STEM; (**Centre**): STEM image; (**Right**): absorbance peak via UV-VIS.

Gold nanoparticles (AuNPs) used in this study were synthesized in endotoxin-free conditions and characterized as described in Materials and Methods. The master batch contained 6 × 10^12^ monodispersed particles/mL of sodium citrate 2.2 mM (in endotoxin-free water) at pH 6.4, corresponding to 0.5 mM Au (0.1 mg/mL) and to a total surface area of 2.6 × 10^15^ nm^2^/mL. AuNPs showed a diameter of 11.8 ± 0.8 nm by STEM, a hydrodynamic diameter of 15.1 ± 3.7 nm by DSL, an absorption peak at 516 nm by UV-VIS, a conductivity of 0.793 mS/cm and a Z-potential of −47.0 ± 5.0 mV. The main NP characteristics are shown in Figure 1.

Evaluation of the endotoxin contamination of AuNPs showed limited contamination (9.0 EU/mg). Since endotoxin could directly activate human monocytes in vitro at concentrations above 0.1 EU/mL [36,39], we used AuNP concentrations with endotoxin contamination below such threshold. After preliminary experiments, we selected 1 µg/mL AuNPs as priming concentration (containing 0.009 EU/mL endotoxin) and the 10-fold higher concentration of 10 µg/mL as challenge (containing 0.09 EU/mL endotoxin). Particles were pre-incubated for 60 min in 50% heat-inactivated human AB serum at 37 °C before being added to cells in culture to allow for the formation of a coating reproducing the naturally occurring biocorona when NPs come in contact with biological fluids. This procedure did not cause particle agglomeration, but instead it promoted monodispersity when added to the culture medium [37,40].

### 3.2. In Vitro Development of Macrophage Innate Memory in 2D vs. 3D Cultures

Purified human blood monocytes were placed in culture either in regular 2D plates or on top of a 3D collagen matrix, as described in Materials and Methods. Cells were either left untreated or exposed for 24 h to LPS or AuNPs. The primary cell activation was measured in terms of production, in the 24 h supernatant, of the inflammatory cytokines TNFα and IL-6, of the chemokine IL-8, and of the anti-inflammatory cytokine IL-1Ra. After supernatant removal and elimination of stimuli, cells were incubated in fresh medium for 6 days, to allow for return to baseline conditions. The 6-day supernatant was removed, and cells were exposed to either fresh medium (control) or LPS for an additional 24 h to assess memory responses, again in terms of cytokine production. 

**Figure 2:**
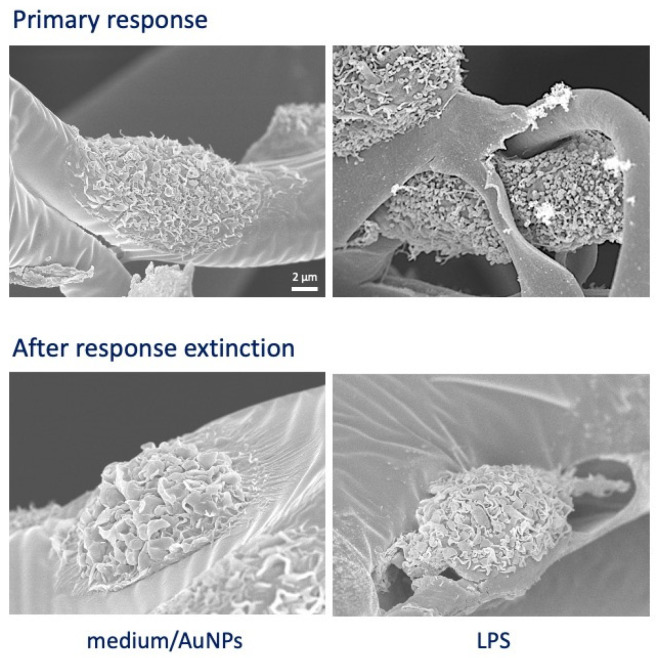
SEM images of monocytes in 3D culture. Upper panels: monocytes exposed for 24 h to culture medium alone or containing AuNPs (showing identical morphology; (**left**)) or LPS (**right**). Lower panels: monocytes primed with either culture medium alone or containing AuNPs (**left**) or LPS (**right**) observed after 6 days of resting in the absence of stimuli.

Cells from six donors were individually tested in parallel in 2D and 3D cultures for their primary and memory responses. SEM images in Figure 2 show the morphology of cells in 3D cultures after 24 h-stimulation with LPS (strong activation; upper right) as compared to cells exposed to medium or AuNPs (upper left) and to unprimed and LPS-primed cells after 6 days of culture (return to quiescent-like morphology; lower panels).

### 3.3. Differences in the Primary Monocyte Reactivity in 2D vs. 3D Cultures

**Figure 3:**
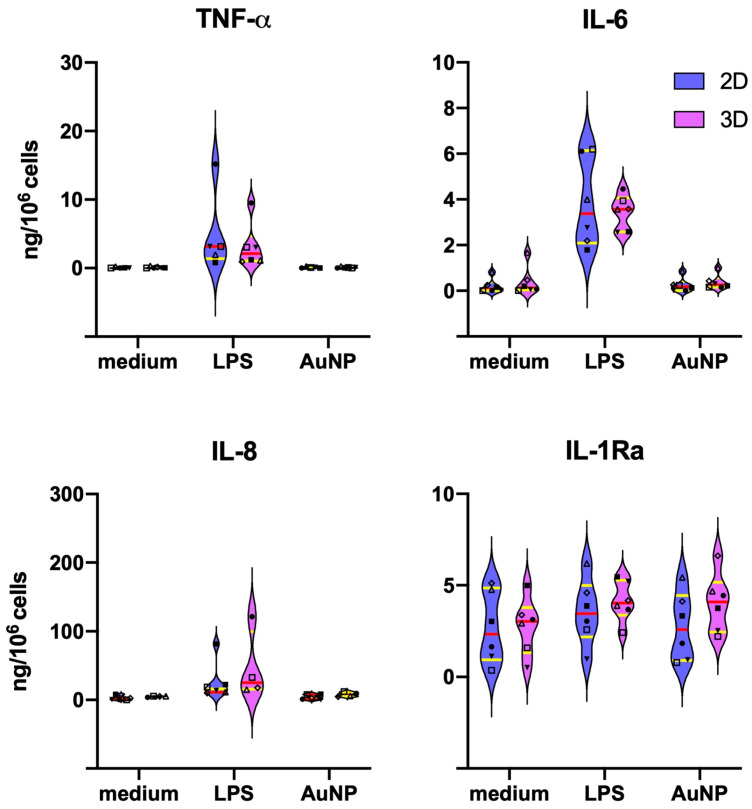
Primary cytokine production by monocytes in 2D vs. 3D culture. Production of the inflammatory cytokines TNFα and IL-6 (**upper panels**), of the chemokine IL-8 (**lower left panel**) and of the anti-inflammatory cytokine IL-1Ra (**lower right panel**) by monocytes of six individual donors stimulated for 24 h with culture medium alone (medium) or containing 1 ng/mL LPS (LPS) or 1 μg/mL AuNPs (AuNP). Parallel 2D (blue) and 3D (purple) cultures were established. Results are expressed in ng/10^6^ cells. The violin plots represent the distribution of the individual donors’ values in each experimental condition. The red line indicates the median value, and the yellow lines the first and third quartiles. Individual donors are represented with different symbols: ◇ donor 1; ■ donor 2; Δ donor 3; ▼ donor 4; ☐ donor 5; ● donor 6. Statistical significance was assessed with the paired one-tailed non-parametric Wilcoxon signed-rank test. LPS stimulation induced a significant increase (*p* < 0.05) of TNFα, IL-6 and IL-8 vs. medium controls. All other differences, including the 2D vs. 3D comparisons, were not significant.

When assessing the capacity of fresh monocytes to react to LPS and AuNPs in terms of cytokine production, no meaningful differences were in general observed between cells in 2D vs. 3D cultures, with some variability observed from donor to donor. Data in Figure 3 show that LPS significantly activates the production of the inflammatory cytokines TNFα and IL-6 and of the chemokine IL-8, while the levels of the anti-inflammatory factor IL-1Ra are already high in unstimulated cells and not substantially changed by LPS stimulation. AuNPs do not show direct activating capacity, in agreement with previous observations [12,28,39]. The individual donors’ data are reported in Table S1.

### 3.4. Extinction of Inflammatory Activation in Macrophages Six Days after Priming 

After the primary stimulation, monocytes were washed to eliminate the residual stimuli and cultured for an additional six days in fresh medium to allow for the extinction of their activation state and return to a quiescent condition. During this period, cells differentiate to macrophages. Their activation state at the end of the resting period was assessed by measuring the spontaneous production of cytokines. As shown in Table S2, the production of the inflammatory cytokines TNFα and IL-6 is essentially zero both in 2D and 3D cultures, independently of the previous priming of cells (with medium, LPS or AuNPs), suggesting that these cells are not any longer in an inflammatory activation state. The production of the chemokine IL-8 is, however, detectable in 3D (but not in 2D cultures) in unprimed/AuNP-primed cells from 4/6 donors and in LPS-primed cells of all donors. LPS-primed cells displayed a generally higher spontaneous IL-8 production as compared to unprimed cells, while AuNP priming did not have substantial effects. Regarding the anti-inflammatory cytokine IL-1Ra, this was detectable at low levels in 2D cultures, while it was easily detectable in all 3D samples. IL-1Ra production was measurable in the unprimed macrophages from all donors at significant levels. Priming with either LPS or AuNPs variably but not substantially modulated the spontaneous production of the anti-inflammatory factor in each donor.

### 3.5. Secondary Response of Unprimed Macrophages

**Figure 4:**
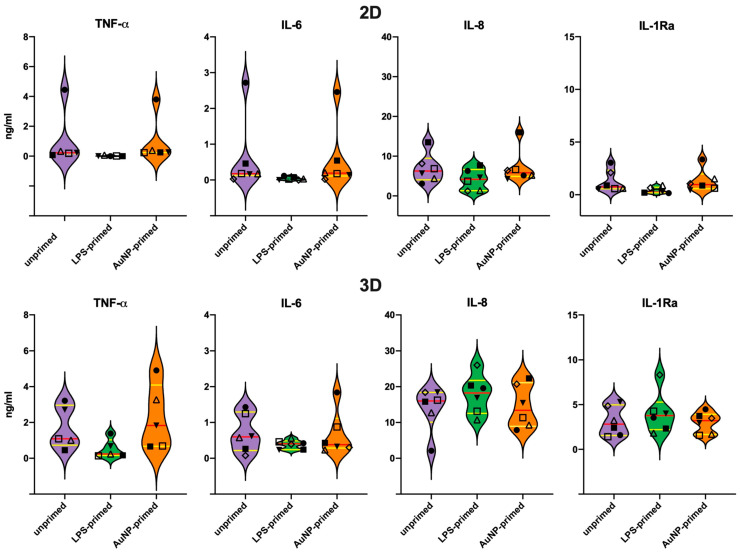
Secondary cytokine production by primed monocytes challenged with LPS in 2D vs. 3D culture. Production of the inflammatory cytokines TNFα and IL-6, the chemokine IL-8 and of the anti-inflammatory cytokine IL-1Ra by monocytes of six donors previously primed in vitro with culture medium alone (unprimed), LPS (LPS-primed) or AuNPs (AuNP-primed). After 24 h priming, cells were rested in culture for 6 days and then challenged with 10 ng/mL LPS for 24 h. Results obtained in 2D cultures are reported in the upper panels, while data from 3D cultures are reported in the lower panels. The violin plots represent the distribution of the individual donors’ values in each experimental condition. The red line indicates the median value, and the yellow lines the first and third quartiles. Individual donors are represented with different symbols: ◇ donor 1; ■ donor 2; Δ donor 3; ▼ donor 4; ☐ donor 5; ● donor 6. Statistical significance was assessed with the paired one-tailed non-parametric Wilcoxon signed-rank test, and show significant differences (*p* < 0.05) between unprimed and LPS-primed production of TNFα (2D and 3D), IL-6 (2D) and IL-1Ra (2D), while the differences between 2D and 3D were significant for IL-8 and IL-1Ra.

The memory response of macrophages that were previously exposed to culture medium alone (unprimed control), LPS or AuNP was examined, after six days of resting, upon a challenge with LPS. Challenge with AuNP was initially assessed as well (data not shown), but it turned out to be ineffective (no activation induced by AuNPs, as already observed in the primary response and in previous studies) [12,28,39], and therefore it was not further pursued. LPS challenge in unprimed control macrophages induced significant production of TNFα, IL-6, and IL-8, which was generally more pronounced in the 3D cultures (Figure 4). Again, the production of IL-1Ra was very low in 2D, while well measurable in 3D cultures. It is notable that, as in the primary response, LPS stimulation had relatively little effect on IL-1Ra production (Figure 4); however, with some donor-dependent variability (compare Tables S2 and S3 for individual data). 

It should be noted that the response of macrophages to LPS, in terms of inflammatory cytokine production, is substantially less abundant compared to monocytes, whereas the quantitative response in terms of IL-8 and IL-1Ra was quite similar between macrophages and monocytes (Figure 4, Tables S1 and S3).

### 3.6. Memory Response of LPS-Primed Macrophages

Compared to unprimed cells, in LPS-primed macrophages, the production of TNFα in response to LPS was significantly decreased both in 2D and 3D cultures, in line with the expected tolerance effect of LPS priming (Figure 4). When looking at the individual data (Table S3), for IL-6 production, tolerance was detectable in 3/6 donors in 3D and in all six in 2D cultures, the overall difference being statistically significant only in the latter case (Figure 4). In the case of the chemokine IL-8, LPS priming did not induce a significant overall effect either in 2D or in 3D (Figure 4), although the individual reactivity varied between donors (Table S3). In 2D cultures, a partial decrease was observed in 2 donors and an increase was detectable in one, while in 3D samples LPS priming had no effect in 5/6 donors and increased the response to challenge in one (donor 6, the same as in 2D cultures) (Table S3). The memory response in terms of the production of the anti-inflammatory cytokine IL-1Ra could be better assessed in 3D cultures (as the IL-1Ra production in 2D cultures was very low) and was donor-dependent, with a partial potentiation in 2 donors, and a decrease in other two. Overall, LPS priming did not induce a significant change in IL-1Ra production (Figure 4). A notable difference between 2D and 3D cultures is in the quantitative production of IL-8 and IL-1Ra, substantially more abundant in 3D cultures, while the production of the inflammatory cytokines TNFα and IL-6 was only slightly higher in 3D cultures.

### 3.7. Memory Response of AuNP-Primed Macrophages

In AuNP-primed macrophages (Figure 4, Table S3), the challenge-induced TNFα production is modulated in a donor-specific fashion, with a difference between 2D and 3D cultures. Thus, while in 2D it seems that AuNP priming does not substantially influence the response to challenge, in 3D cultures a partial decrease of response is evident in 3 donors. A very similar picture can be observed for the production of the other inflammatory cytokine, IL-6, with a general lack of effect in 2D samples, whereas in 3D cultures two donors show partial potentiation and one partial tolerance. In the case of the chemokine IL-8, the results in 2D and 3D cultures were superimposable, with no substantial effect due to AuNP priming except for one donor (donor 6), in which priming induced a clear potentiation of the memory response. For the anti-inflammatory factor IL-1Ra, better detectable in 3D, AuNP priming could induce a partial tolerance response in one donor, a potentiated response in another one, and no significant changes in the others. Again, the memory effect seems to strongly depend on the donor.

## 4. Discussion

This study aimed at assessing whether human monocyte/macrophage reactivity in vitro can differ depending on the culture conditions. In particular, we compared conventional 2D cultures on flat plastic surfaces to 3D cultures in a biological matrix of collagen, derived from decellularized bovine skin and providing a mesh of collagen fibrils with pores around 100 μm. This matrix is used in clinical settings as dermal/epidermal substitute for repairing ulcers and was reportedly able to promote M2/healing macrophage phenotype, i.e., the default phenotype of resident macrophages in homeostatic conditions [40]. Cells seeded in the 3D matrix survived and differentiated as expected, and their sensitivity to activation stimuli was evident also from the morphological point of view (Figure 2). We used the two culture systems in comparison to assess the reactivity of human monocytes/macrophages to AuNPs, using the bacterial LPS as an activation benchmark and examining two important innate immune functions, i.e., the immediate inflammation-related response to the stimulation of fresh monocytes and the secondary reaction of monocyte-derived macrophages after previous priming (innate memory response). The results show that the primary response is not significantly different between 2D and 3D cultures (Figure 3) and that, in agreement with previous results [12,28,39], AuNPs do not induce a significant cell activation, opposite to the substantial induction of TNFα, IL-6 and IL-8 production in response to LPS. On the other hand, the production of the anti-inflammatory factor IL-1Ra is detectable at measurable levels in unstimulated cells and is not significantly varied in cells exposed to LPS or AuNPs. It is known that LPS can induce IL-1Ra production at later times as a feedback mechanism to shut down inflammation. Our data show a slight tendency towards increased IL-1Ra production to LPS, and also to AuNPs in 3D cultures, which, however, did not reach statistical significance due to the substantial donor-to-donor variability. Indeed, monocytes from some donors showed a clear increase while others did not, implying different response kinetics depending on the individual conditions.

While it is now clear that many NPs do not have the capacity for activating human monocytes if they are endotoxin-free [12,13,35,36,39], some data point to the possibility that they can prime monocytes and induce innate memory, which then changes the secondary reaction of cells to a subsequent stimulation [28,29,30,31,32]. We have examined the capacity of AuNPs to induce innate memory in monocytes from six different donors in 2D and 3D cultures. Innate memory is an important phenomenon of immune reprogramming that allows innate cells to react is a more efficient way to a challenge if previously exposed to foreign/dangerous agents [14,15,16,17,18,19,20]. The results show that AuNPs do not have a significant capacity to induce memory in 2D cultures, with unprimed vs. AuNP-primed cells from all donors showing a comparable response to LPS (Figure 4, Table S3). In 3D cultures, although the global response is never different between unprimed and AuNP-primed cells, when examining the response of individual donors, it is possible to see memory effects, as for instance for Donor 3 that shows an AuNP priming-dependent decrease in TNFα, IL-6, and IL-1Ra production, which is not evident in 2D cultures, or for Donor 6 that shows an AuNP priming-dependent increase in IL-8 and IL-1Ra that was undetectable in 2D cultures.

Two important notions should be underlined here. First, the unstimulated production of IL-8 and IL-1Ra is detectable at higher levels in unprimed/primed macrophages in 3D cultures compared to the 2D cultures, whereas the production of the inflammatory cytokines TNFα and IL-6 is essentially undetectable in both conditions (Table S2). This implies that the 3D cultures provide a more physiological microenvironment, which promotes the homeostatic tissue-like functions of macrophages, characterised by lack of production of inflammatory factors and abundant production of anti-inflammatory factors [7,8,41]. The significant production of IL-8 goes in the same direction, as this chemokine is involved in ensuring the appropriate homeostatic trafficking of neutrophils and other cells in non-inflammatory conditions [42,43,44]. Thus, in 3D cultures monocytes developed in the direction of M2/healing macrophages by adopting a classical tissue resident-like anti-inflammatory and homeostatic functional phenotype. Notably, IL-1Ra is detectable at significantly lower levels in macrophage 2D cultures, a finding that supports the suitability of 3D cultures for a more realistic assessment of macrophage reactivity. However, it should be noted that we could not find significant qualitative differences in the innate memory activities between cells cultured in 2D vs. 3D conditions. On the other hand, as already mentioned, there is no substantial qualitative and quantitative difference in the reactivity of fresh monocytes in 2D vs. 3D cultures, suggesting that 2D cultures are fully suitable for evaluating acute innate responses.

The second important finding is that the memory response to AuNPs is strongly dependent on donors. While memory responses induced by priming with LPS (a very strong stimulus) are clearer, with exposure to AuNPs (a stimulus that does not induce a detectable primary response) it becomes evident that the priming capacity substantially depends on the donor. Thus, as an example, the 3D memory response in terms of IL-6 is increased production in two donors, a decrease in another two, and no change in the last two donors. This is fully in line with previous preliminary observations [28,30] and underlines the importance of ‘immunobiography”, i.e., the history of exposure and challenges experienced by the immune system, which is unique for each of us and that can significantly bias our capacity to react appropriately to new challenges [33]. Although the number of donors in this study is limited (six donors), they are quite homogeneous in terms of age and health status (young, healthy, no medications, non-smokers). They encompass individual of both sexes and of two different ethnic groups, but neither parameter is associated with a defined type of response, further suggesting that the individual history of exposure may be at the basis of response variability.

These findings suggest some important issues in directing future research: the need for adopting suitable models for our in vitro studies of human innate memory, and the importance of performing individual assessments (precision medicine, precision diagnosis, precision toxicology), since it is not possible to generalize findings of efficacy or toxicity to a population that is widely different also in terms of immune history.

## Data Availability

The data presented in this study are available in this article and its supplementary material.

## Supplementary Materials

The following are available online at https://www.mdpi.com/2079-4991/11/1/207/s1, Table S1: Primary cytokine production by monocytes in 2D vs. 3D cultures; Table S2: Baseline cytokine production by unprimed and primed monocytes after 6-day resting in 2D vs. 3D cultures; Table S3: Secondary cytokine production by unprimed and primed monocytes challenged with LPS after 6-day resting in 2D vs. 3D cultures.

## References

[1] Dobrovolskaia M.A., Shurin M., Shvedova A.A. (2016). Current understanding of interactions between nanoparticles and the immune system. Toxicol. Appl. Pharmacol..

[2] Fadeel B. (2019). Hide and seek: Nanomaterial interactions with the immune system. Front. Immunol..

[3] Boraschi D., Italiani P., Palomba R., Decuzzi P., Duschl A., Fadeel B., Moghimi S.M. (2017). Nanoparticles and innate immunity: New perspectives on host defence. Sem. Immunol..

[4] Medzhitov R., Janeway C.A. (1997). Innate immunity: The virtues of a non-clonal system of recognition. Cell.

[5] Carta S., Yassi S., Pettinati I., Delfino L., Dinarello C.A., Rubartelli A. (2011). The rate of IL-1β secretion in different myeloid cells varies with the extent of redox response to TLR triggering. J. Biol. Chem..

[6] Italiani P., Boraschi D. (2014). From monocytes to M1/M2 macrophages: Phenotypical vs. functional differentiation. Front. Immunol..

[7] Mills C.D., Lenz L.L., Ley K. (2015). M1/M2 Macrophages: The Arginine Fork in the Road to Health and Disease.

[8] Holst O., Ulmer A.J., Brade H., Flad H.D., Rietschel E.T. (1996). 1996. Biochemistry and cell biology of bacterial endotoxins. FEMS Immunol. Med. Microbiol..

[9] Mestas J., Hughues C.C.W. (2004). Of mice and not men: Differences between mouse and human immunology. J. Immunol..

[10] Davis M.M. (2008). A prescription for human immunology. Immunity.

[11] Italiani P., Mazza E.M.C., Lucchesi D., Cifola I., Gemelli C., Grande A., Battaglia C., Bicciato S., Boraschi D. (2014). Transcriptomic profiling of the development of the inflammatory response in human monocytes in vitro. PLoS ONE.

[12] Li Y., Italiani P., Casals E., Valkenborg D., Mertens I., Baggerman G., Nelissen I., Puntes V., Boraschi D. (2016). Assessing the immunosafety of engineered nanoparticles with a novel in vitro model based on human primary monocytes. ACS Appl. Mater. Interfaces.

[13] Ferretti A.M., Usseglio S., Mondini S., Drago C., La Mattina R., Chini B., Verderio C., Leonzino M., Cagnoli C., Joshi P. (2020). Towards bio-compatible magnetic nanoparticles: Immune-related effects, in vitro internalization, and in vivo bio-distribution of zwitterionic ferrite nanoparticles with unexpected renal clearance. J. Colloid Interface Sci..

[14] Beeson P.B. (1946). Development of tolerance to typhoid bacterial pyrogen and its abolition by reticulo-endothelial blockade. Proc. Soc. Exp. Biol. Med..

[15] Howard J.G., Biozzi G., Halpern B.N., Stiffel C., Mouton D. (1959). The effect of *Mycobacterium tuberculosis* (BCG) infection on the resistance of mice to bacterial endotoxin and *Salmonella enteritidis* infection. Br. J. Exp. Pathol..

[16] Bistoni F., Vecchiarelli A., Cenci E., Puccetti P., Marconi P., Cassone A. (1986). Evidence for macrophage-mediated protection against lethal *Candida albicans* infection. Infect. Immun..

[17] Netea M.G., Quintin J., van der Meer J.W.M. (2011). Trained immunity: A memory for innate host defense. Cell Host Microbe.

[18] Milutinovi’c B., Kurtz J. (2016). Immune memory in invertebrates. Sem. Immunol..

[19] Cooper D., Eleftherianos I. (2017). Memory and specificity in the insect immune system: Current perspectives and future challenges. Front. Immunol..

[20] Gourbal B., Pinaud S., Beckers G.J.M., Van Der Meer J.W.M., Conrath U., Netea M.G. (2018). Innate immune memory: An evolutionary perspective. Immunol. Rev..

[21] Arts R.J.W., Joosten L.A.B., Netea M.G. (2018). The potential role of trained immunity in autoimmune and autoinflammatory disorders. Front. Immunol..

[22] Salam A.P., Borsini A., Zunszain P.A. (2018). Trained innate immunity: A salient factor in the pathogenesis of neuroimmune psychiatric disorders. Mol. Psychiatry.

[23] Salani F., Sterbini V., Sacchinelli E., Garramone M., Bossu P. (2019). Is innate memory a double-edge sword in Alzheimer’s Disease? A reappraisal of new concepts and old data. Front. Immunol..

[24] Töpfer E., Boraschi D., Italiani P. (2015). Innate immune memory: The latest frontier of adjuvanticity. J. Immunol. Res..

[25] Xing Z., Afkhami S., Bavananthasivam J., Fritz D.K., D’Agostino M.R., Vaseghi-Shanjani M., Yao Y., Jeyanathan M. (2020). Innate immune memory of tissue-resident macrophages and trained innate immunity: Re-vamping vaccine concept and strategies. J. Leukocyte Biol..

[26] Sánchez-Ramón S., Conejero L., Netea M.G., Sancho D., Palomares Ó., Subiza J.L. (2018). Trained immunity-based vaccines: A new paradigm for the development of broad-spectrum anti-infectious formulations. Front. Immunol..

[27] Mulder W.J.M., Ochando J., Joosten L.A.B., Fayad Z.A., Netea M.G. (2019). Therapeutic targeting of trained immunity. Nat. Rev. Drug Discov..

[28] Italiani P., Boraschi D. (2017). Induction of innate immune memory by engineered nanoparticles: A hypothesis that may become true. Front. Immunol..

[29] Lebre F., Boland J.B., Gouveia P., Gorman A., Lundahl M., O’Brien F.J., Coleman J., Lavelle E.C. (2020). Pristine graphene induces innate immune training. Nanoscale.

[30] Swartzwelter B.J., Barbero F., Verde A., Mangini M., Pirozzi M., De Luca A.C., Puntes V.F., Leite L.C.C., Italiani P., Boraschi D. (2020). Gold nanoparticles modulate BCG-induced innate iummune memory in nhuman monocytes by shifting the memory response towards tolerance. Cells.

[31] Auguste M., Balbi T., Ciacci C., Canonico B., Papa S., Borello A., Vezzulli L., Canesi L. (2020). Shift in immune parameters after repeated exposure to nanoplastics in the Marine Bivalve *Mytilus*. Front. Immunol..

[32] Italiani P., Della Camera G., Boraschi D. (2020). Induction of innate immune memory by engineered nanoparticles in monocytes/macrophages: From hypothesis to reality. Front. Immunol..

[33] Franceschi C., Salvioli S., Garagnani P., de Eguileor M., Monti D., Capri M. (2017). Immunobiography and the heterogeneity of immune responses in the elderly: A focus on inflammaging and trained immunity. Front. Immunol..

[34] Ojea-Jiménez I., Bastús N.G., Puntes V. (2011). Influence of the sequence of the reagents addition in the citrate-mediated synthesis of gold nanoparticles. J. Phys. Chem. C.

[35] Li Y., Italiani P., Casals E., Tran N., Puntes V.F., Boraschi D. (2015). Optimising the use of commercial LAL assays for the analysis of endotoxin contamination in metal colloids and metal oxide nanoparticles. Nanotoxicology.

[36] Oostingh G.J., Casals E., Italiani P., Colognato R., Stritzinger R., Ponti J., Pfaller T., Kohl Y., Ooms D., Favilli F. (2011). Problems and challenges in the development and validation of human cell-based assays to determine nanoparticle-induced immunomodulatory effects. Particle Fibre Toxicol..

[37] Piella J., Bastús N.G., Puntes V. (2017). Size-dependent protein-nanoparticle interaction in citrate-stabilized gold nanoparticles: The emergence of the protein corona. Bioconj. Chem..

[38] Comenge J., Sotelo C., Romero F., Gallego O., Barnadas A., Garcia-Caballero Parada T., Dominguez F., Puntes V.F. (2012). Detoxifying antitumoral drugs via nanoconjugation: The case of gold nanoparticles and cisplatin. PLoS ONE.

[39] Li Y., Shi Z., Radauer-Preiml I., Andosch A., Casals E., Luetz-Meidl U., Cobaleda M., Lin Z., Jaberi-Douraki M., Italiani P. (2017). Bacterial endotoxin (LPS) binds to the surface of gold nanoparticles, interferes with biocorona formation and induces human monocyte inflammatory activation. Nanotoxicology.

[40] Montanaro M., Meloni M., Anemona L., Giurato L., Scimeca M., Izzo V., Servadei F., Smirnov A., Candi E., Mauriello A. (2020). Macrophage activation and M2 polarization in wound bed of diabetic patients treated by dermal/epidermal substitute Nevelia. Int. J. Lower Extr. Wounds.

[41] Röszer T. (2015). Understanding the misterious M2 macrophage through activation markers and effector mechanisms. Med. Inflamm..

[42] Sherwood J., Bertrand J., Nalesso G., Poulet B., Pitsillides A., Brandolini L., Karystinou A., De Bari C., Luyten F.P., Pitzalis C. (2015). A homeostatic function of CXCR2 signalling in articular cartilage. Ann. Rheum. Dis..

[43] Nicolás-Ávila J.A., Adrover J.M., Hidalgo A. (2017). Neutrophils in homeostasis, immunity and cancer. Immunity.

[44] Pekalski M.L., Rubio Garcia A., Ferreira R.C., Rainbow D.B., Smyth D.J., Mashar M., Brady J., Savinykh N., Castro Dopico X., Mahmood S. (2017). Neonatal and adult recent thymic emigrants produce IL-8 and express complement receptors CR1 and CR2. JCI Insight.

