# Supplementary material for: Interaction between Macrophages and Nanoparticles: In Vitro 3D Cultures for the Realistic Assessment of Inflammatory Activation and Modulation of Innate Memory
Source: Nanomaterials (Basel). 2021 Jan 15;11(1):207. doi: 10.3390/nano11010207 (PMC7830034; doi:10.3390/nano11010207)
Supplement: Supplementary file 1 [file nanomaterials-11-00207-s001.pdf]

nanomaterials

Article

Interaction between Macrophages and Nanoparticles: In Vitro 3D Cultures for the Realistic Assessment of Inflammatory Activation and Modulation of Innate Memory

Benjamin J. Swartzwelter [1,†], Alessandro Verde [1], Laura Rehak [2], Mariusz Madej [1,‡], Victor. F. Puntes [3], Anna Chiara De Luca [1], Diana Boraschi [1,4,*] and Paola Italiani [1,4,*]

1 Institute of Biochemistry and Cell Biology, National Research Council, 80131 Napoli, Italy; swartzwe@colorado.edu (B.J.S.); alessandro.verde@ibbc.cnr.it (A.V.); mariusz.madej@ocello.nl (M.M.); annachiara.deluca@ibbc.cnr.it (A.L.D.L.)
2 Athena Biomedical Innovations, 00100 Roma, Italy; laurarehak@gmail.com
3 Institut Català de Nanociència i Nanotecnologia (ICN2), CSIC and The Barcelona Institute of Science and Technology (BIST), Campus UAB, 08193 Bellaterra, Barcelona, Spain; victor.puntes@icn2.cat
4 Stazione Zoologica Anton Dohrn, 80121 Napoli, Italy
* Correspondence: diana.boraschi@ibbc.cnr.it (D.B.); paola.italiani@ibbc.cnr.it (P.I.)
† Current address: Department of Microbiology, Immunology and Pathology, Colorado State University, Fort Collins, CO 80521, USA.
‡ Current address: OcellO B.V., 2333 CH Leiden, The Netherlands.

Table S1. Primary cytokine production by monocytes in 2D vs. 3D cultures.

Culture conditions	Cytokine	Cytokine production[1]					
		Donor 1	Donor 2	Donor 3	Donor 4	Donor 5	Donor 6
**2D**							
medium	TNFα	nt[2]	0.06 (0.10)	0.21 (0.02)	0	0	0
LPS		nt	0.82 (0.28)	1.95 (0.53)	3.15 (0.31)	3.15 (0.11)	15.17 (0.76)
AuNP		nt	0.02 (0.01)	0.26 (0.03)	0.12 (0.02)	0.09 (0.09)	0
medium	IL-6	0.24 (0.02)	0.15 (0.04)	0.81 (0.09)	0	0	0
LPS		2.19 (0.19)	1.78 (0.31)	3.99 (0.23)	2.75 (0.12)	6.20 (2.41)	6.11 (0.19)
AuNP		0.25 (0.07)	0.12 (0.03)	0.85 (0.11)	0	0.23 (0.25)	0
medium	IL-8	2.43 (0.08)	8.15 (1.70)	3.65 (0.02)	0.58 (0.14)	0.08 (0.11)	0.60 (0.02)
LPS		10.11 (7.50)	22.38 (3.40)	11.55 (0.80)	13.88 (1.77)	18.75 (4.84)	81.85 (9.56)
AuNP		2.41 (0.01)	8.46 (2.40)	3.85 (0.07)	0.13 (0.03)	7.97 (6.72)	1.01 (0.42)
medium	IL-1Ra	5.12 (0.85)	3.04 (0.44)	4.77 (0.95)	1.13 (0.11)	0.37 (0.08)	1.66 (0.12)
LPS		4.59 (0.32)	3.88 (1.05)	6.20 (1.04)	0.98 (0.01)	2.60 (0.17)	3.05 (0.21)
AuNP		4.12 (1.13)	3.35 (0.59)	5.43 (0.40)	0.94 (0.03)	0.79 (0.17)	1.85 (0.28)
**3D**							
medium	TNFα	0.15 (0.02)	0.05 (0.01)	0.30 (0.01)	0	0	0
LPS		0.93 (0.13)	1.21 (0.18)	1.11 (0.18)	3.02 (0.13)	3.06 (0.54)	9.50 (0.44)
AuNP		0	0.04 (0.01)	0.25 (0.04)	0.07 (0.01)	0.09 (0.08)	0
medium	IL-6	0.47 (0.04)	0.19 (0.06)	1.66 (0.06)	0.05 (0.01)	0	0.06 (0.04)
LPS		3.57 (0.11)	2.58 (0.67)	3.56 (0.22)	2.55 (0.02)	3.93 (1.68)	4.45 (0.60)
AuNP		0.42 (0.19)	0.21 (0.08)	1.00 (0.09)	0.30 (0.03)	0.15 (0.15)	0.15 (0.09)
medium	IL-8	5.14 (0.10)	nt	5.61 (0.18)	nt	5.35 (1.58)	3.89 (1.50)
LPS		17.71 (1.70)	nt	15.26 (0.27)	nt	32.88 (11.08)	121.55 (21.1)
AuNP		4.74 (0.04)	nt	5.85 (0.07)	nt	12.00 (2.37)	9.60 (2.20)
medium	IL-1Ra	3.39 (0.53)	4.99 (1.06)	2.94 (0.99)	0.52 (0.02)	1.60 (0.34)	3.14 (1.14)
LPS		4.17 (0.60)	5.47 (0.28)	3.90 (0.23)	5.22 (1.20)	2.43 (0.66)	3.70 (1.04)
AuNP		6.62 (1.66)	3.75 (1.11)	4.68 (2.39)	2.53 (0.20)	2.22 (0.44)	4.44 (1.14)

[1] Cytokines were measured in the supernatant of 2D and 3D monocyte cultures exposed to culture medium alone for 24 h. Results are the mean values of 2-8 replicate determinations and are expressed in ng/10^6 cells. SD is shown in parentheses. Undetectable cytokine levels are shown as zero.

[2] nt = not tested

nanomaterials

MDPI

Table S2. Baseline cytokine production by unprimed and primed monocytes after 6-day resting in 2D vs. 3D cultures.

Cytokine	Cytokine production[1]																	
	Unprimed						LPS-primed						AuNP-primed					
	D1	D2	D3	D4	D5	D6	D1	D2	D3	D4	D5	D6	D1	D2	D3	D4	D5	D6
**2D**																		
TNFα	0	0	0	0	0	0	0	0	0	0	0	0	0	0	0	0	0	0
IL-6	0	0	0	0	0	0	0	0	0	0	0	0	0	0	0	0	0	0
IL-8	0	0	0	0	0	0	0.14 (0.08)	0	0	0	0.17 (0.07)	0	0	0	0	0	0.08 (0.02)	0
IL-1Ra	0.99 (0.72)	0.70 (0.17)	0.49 (0.15)	0.29 (0.01)	0.27 (0.11)	1.07 (0.19)	0.36 (0.10)	0.32 (0.16)	0.37 (0.44)	0.29 (0.03)	0.37 (0.48)	0.09 (0.02)	0.46 (0.11)	0.63 (0.10)	1.18 (0.86)	0.47 (0.03)	0.17 (0.06)	1.41 (0.63)
**3D**																		
TNFα	0	0	0	0	0	0	0	0	0	0	0	0	0	0	0	0	0	0
IL-6	0	0	0	0	0	0	0	0	0.07 (0.01)	0	0	0	0	0	0	0	0	0
IL-8	2.91 (0.35)	0.65 (0.18)	2.08 (0.05)	0.58 (0.07)	0	0	3.48 (0.15)	2.46 (0.27)	3.09 (0.28)	2.70 (0.80)	0.29 (0.04)	2.50 (1.59)	1.38 (0.08)	1.87 (0.39)	2.07 (0.10)	0.76 (0.30)	0	0
IL-1Ra	4.17 (0.39)	0.74 (0.44)	0.67 (0.18)	2.67 (2.00)	0.77 (0.32)	2.33 (0.82)	1.59 (0.30)	1.33 (0.04)	1.37 (1.10)	4.19 (1.46)	1.32 (0.33)	5.74 (3.38)	0.97 (0.46)	1.85 (1.30)	1.50 (0.23)	3.72 (1.82)	0.53 (0.19)	4.86 (0.12)

[1] Cytokines were measured in the supernatant of 2D and 3D monocyte cultures that were previously primed for 24 h with culture medium alone (unprimed) or containing LPS (1 ng/mL) or AuNP (1 µg/mL), cultured with culture medium alone for 6 additional days, and eventually exposed for further 24 h to fresh culture medium. Results are the mean values of 2-4 replicate determinations and are expressed in ng/10^6 cells. SD is shown in parentheses. Undetectable cytokine levels are shown as zero.

Table S3. Secondary cytokine production by unprimed and primed monocytes challenged with LPS after 6-day resting in 2D vs. 3D cultures.

Cytokine	Cytokine production[1]																	
	Unprimed						LPS-primed						AuNP-primed					
	D1	D2	D3	D4	D5	D6	D1	D2	D3	D4	D5	D6	D1	D2	D3	D4	D5	D6
**2D**																		
TNFα	nt[2]	0.08 (0.02)	0.33 (0.02)	0.26 (0.05)	0.19 (0.07)	4.45 (0.19)	nt	0	0.07 (0.02)	0	0.02 (0.01)	0	nt	0.26 (0.06)	0.39 (0.09)	0.26 (0.16)	0.23 (0.03)	3.80 (0.32)
IL-6	0.03 (0.01)	0.46 (0.11)	0.17 (0.03)	0.16 (0.01)	0.18 (0.07)	2.72 (0.09)	0	0.08 (0.01)	0.03 (0.01)	0	0.03 (0.01)	0.12 (0.08)	0.02 (0.01)	0.54 (0.07)	0.21 (0.04)	0.14 (0.11)	0.18 (0.04)	2.46 (0.08)
IL-8	8.15 (1.10)	13.49 (2.72)	4.38 (0.26)	5.67 (1.19)	6.86 (4.97)	3.10 (0.79)	1.23 (0.47)	7.71 (2.08)	1.35 (0.13)	4.64 (1.74)	3.72 (1.45)	6.30 (1.67)	6.42 (0.69)	15.99 (2.15)	5.22 (1.43)	4.25 (0.41)	6.64 (0.83)	5.15 (3.03)
IL-1Ra	2.10 (1.05)	0.88 (0.20)	0.61 (0.23)	0.52 (0.10)	0.58 (0.16)	3.04 (0.46)	0.66 (0.09)	0.20 (0.03)	0.88 (0.11)	0.27 (0.11)	0.23 (0.38)	0.15 (0.06)	1.05 (0.14)	0.87 (0.10)	1.52 (0.68)	0.43 (0.20)	0.62 (0.2s2)	3.35 (0.92)
**3D**																		
TNFα	nt	0.45* (0.22)	1.01* (0.06)	2.71* (0.28)	1.09* (0.32)	3.22* (0.30)	nt	0.16* (0.12)	0.24* (0.02)	0.68* (0.09)	0.14* (0.02)	1.38* (0.12)	nt	0.66* (0.15)	0.33 (0.08)	1.83* (0.60)	0.68* (0.06)	4.91 (1.54)
IL-6	0.08 (0.05)	0.26 (0.16)	0.58* (0.04)	0.62* (0.07)	1.25* (0.52)	1.43* (0.03)	0.39 (0.32)	0.24 (0.15)	0.57* (0.24)	0.24* (0.10)	0.46* (0.05)	0.42* (0.04)	0.30* (0.12)	0.43 (0.11)	0.24 (0.07)	0.33 (0.14)	0.87* (0.06)	1.84 (0.63)
IL-8	18.41* (5.40)	15.81 (7.33)	12.77* (2.14)	18.44* (1.09)	16.24* (3.88)	2.11 (1.27)	26.03* (6.65)	20.34* (5.80)	10.77* (0.86)	16.90* (0.95)	13.16* (1.92)	19.60* (8.83)	20.70* (2.82)	22.34* (2.34)	9.29* (0.45)	15.47* (0.53)	11.34* (0.45)	7.88 (3.70)
IL-1Ra	4.86* (3.01)	2.42* (0.97)	3.23* (0.60)	5.30* (0.32)	1.42* (0.38)	1.59* (0.43)	8.32*v* (4.73)	2.33* (1.50)	1.82* (0.80)	4.00* (0.65)	4.27* (0.72)	3.54* (0.52)	3.47* (0.37)	3.73* (0.34)	1.69* (0.27)	2.96* (2.19)	1.57* (0.79)	4.47 (3.20)

[1] Cytokines were measured in the supernatant of 2D and 3D monocyte cultures that were previously primed for 24 h with culture medium alone (unprimed), LPS (1 ng/mL) or AuNP (1 µg/mL), then cultured with fresh medium for 6 additional days, and eventually challenged with LPS (10 ng/mL) for 24 h. Results are the mean values of 2-4 replicate determinations and are expressed in ng/10^6 cells. SD is shown in parentheses. Undetectable cytokine levels are shown as zero.

nt = not tested.

* $p < 0.05$ between 2D and 3D
